# Multiplicative Effects of Essential Oils and Other Active Components on Skin Tissue and Skin Cancers

**DOI:** 10.3390/ijms25105397

**Published:** 2024-05-15

**Authors:** Hyeong Jae Kim, Jeong Hee Hong

**Affiliations:** Department of Physiology, College of Medicine, Gachon University, Lee Gil Ya Cancer and Diabetes Institute, 155 Getbeolro, Yeonsu-gu, Incheon 21999, Republic of Korea; lilili1125@naver.com

**Keywords:** essential oils, skin homeostasis, inflammation, skin diseases, melanoma

## Abstract

Naturally derived essential oils and their active components are known to possess various properties, ranging from anti-oxidant, anti-inflammatory, anti-bacterial, anti-fungal, and anti-cancer activities. Numerous types of essential oils and active components have been discovered, and their permissive roles have been addressed in various fields. In this comprehensive review, we focused on the roles of essential oils and active components in skin diseases and cancers as discovered over the past three decades. In particular, we opted to highlight the effectiveness of essential oils and their active components in developing strategies against various skin diseases and skin cancers and to describe the effects of the identified essential-oil-derived major components from physiological and pathological perspectives. Overall, this review provides a basis for the development of novel therapies for skin diseases and cancers, especially melanoma.

## 1. Introduction

Essential oils (EO)s are natural oils secreted as secondary metabolites or concentrated plant extracts from many parts of aromatic plants (especially the bark, fruits, and flowers) [1]. EOs and their active components possess many biological properties, including anti-bacterial, anti-virus, anti-fungal, anti-inflammatory, and anti-cancer properties [2,3]. The skin is the first barrier that protects against external stimuli and microorganisms. Moreover, the skin possesses unique characteristics, such as its stratified structure, various cell types, and the pigmentation process, which are distinct from other tissues. Notably, EOs are used to treat several diseases, including skin diseases such as eczema, psoriasis, dermatitis, and skin cancers. In this review, we aimed to highlight the benefits of various EOs and several plant oils (POs) in terms of skin reactivity and the treatment of skin cancers, especially melanoma, with a PubMed-based selection of studies published in the last three decades which address the effects of EOs/POs on skin diseases and skin cancers. Overall, a summary of the effectiveness of EOs would help alleviate skin diseases and promote the development of therapeutic strategies against various skin diseases and skin cancers.

## 2. Anti-Inflammatory Role of EOs

### 2.1. Citrus limetta Peel Essential Oil (Cl-EO)

*Citrus limetta* (*C. limetta*) Risso peels exhibit various medicinal activities, such as anti-oxidant and anti-inflammatory activities [4], due to the presence of large quantities of flavonoids [5]. EO, a widely used ingredient in cosmetic and pharmaceutical products, is an important product of citrus fruit peels [6,7]. EOs and their constituents from citrus fruits exhibit anti-inflammatory activities in vitro by inhibiting the biosynthesis of inflammatory cytokines [8]. Inflammatory cytokines, such as tumor necrosis factor (TNF)-α, interleukin (IL)-1β, and IL-6, are found to be dose-dependently reduced by treatment with *C. limetta* peel EO (Cl-EO) in the lipopolysaccharide (LPS)-exposed primary macrophages or the 12-O-tetradecanoylphorbol-13-acetate (TPA), used as protein kinase C (PKC) activator, -exposed mouse ear [9]. In addition, Cl-EO is found to inhibit, dose-dependently, oxidative stress in hydrogen peroxide (H_2_O_2_)-exposed primary macrophages and TPA-exposed mice [9].

### 2.2. Baccharis dracunculifolia Essential Oil (BD-EO)

*Baccharis dracunculifolia (B. dracunculifolia*) is a domestic plant which is widely used as an immunomodulator and an anti-bacterial and anti-diabetic agent, but it possesses many other properties [10,11]. Several studies have evaluated the medicinal actions of *B. dracunculifolia*, especially its anti-microbial effects [12,13,14]. Dos Santos et al. reported the anti-inflammatory effects of *B. dracunculifolia* leaves extract on a paw edema model, which is induced by carrageenan and formalin [15]. Additionally, *B. dracunculifolia* leaf extract is found to exhibit an inhibitory effect on LPS-challenged murine macrophages by inhibiting the biosynthesis of IL-6 and IL-10 [16,17]. Ear edema, infiltration, and proliferation/differentiation of keratinocytes, as well as the activities of myeloperoxidase and N-acetylglutamate synthase, are downregulated by BD-EO administration in a TPA-induced acute and chronic inflammatory mouse model [18].

### 2.3. Perilla frutescens L. Britt Essential oil (PF-EO)

**Figure 1:**
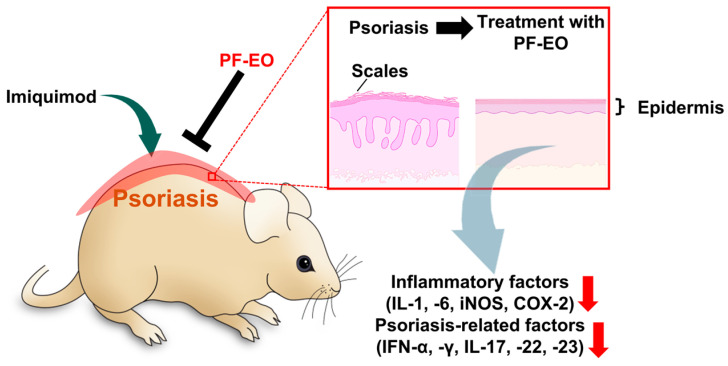
Schematic of the alleviation effect of PF-EO treatment in an imiquimod-mediated mouse model of psoriasis. PF-EO treatment induces the downregulation of psoriasis symptoms, such as erythema, scaling, and epidermal thickening, and the expression of inflammatory factors and psoriasis-related factors [22]. The red arrow indicates downregulated expression in the epidermis of a PF-EO-treated mouse. PF-EO: *Perilla frutescens* L. Britt essential oil; IL: interleukin; iNOS: inducible nitric oxide synthase; COX-2: cyclooxygenase-2; IFN: interferon.

*Perilla frutescens* (*P. frutescens*) L. Britt is a herbaceous plant that plays a critical role in Chinese medicine and is used to treat various pathological symptoms, including abdominal pain, nausea, cold, constipation, food poisoning, and cough [19]. The EOs from *P. frutescens* have been noted to possess anti-bacterial, anti-inflammatory, and anti-oxidant properties [20,21]. In addition, psoriasis symptoms, such as erythema, scaling, and epidermal thickening, have previously found to be alleviated by treatment with *P. frutescens* L. Britt EO (PF-EO) in a mouse model of imiquimod (IMQ)-induced psoriasis [22]. Inflammation-related factors, such as inducible nitric oxide synthase (iNOS), cyclooxygenase (COX)-2, IL-1, IL-6, and neutrophil proliferation, are downregulated by PF-EO treatment in the full skin and epidermis of an IMQ-induced psoriasis in mouse [22]. IMQ induces dendritic cell activation, which is related to the activation of macrophages and T-lymphocytes [23,24,25]. The upregulated mRNA expression levels of IL-17, IL-22, IL-23, interferon (IFN)-α, and IFN-γ due to IMQ administration are found to be reduced by PF-EO treatment in the skin [22]. The production of psoriasis-development-associated cytokines, such as IL-1, IL-6, IL-17, IL-23, and nuclear factor kappa B (NF-κB), are downregulated by PF-EO treatment in the serum of an IMQ-induced mouse [22] (Figure 1).

### 2.4. Grapefruit Essential Oil (G-EO)

Citrus EO exhibits various biological activities, such as anti-microbial [26], anti-oxidant [27], and anti-fungal activities [28]. Grapefruit (*Citrus maxima* (Burm). Merr) is one of the citrus producers, and the major components of grapefruit EO (G-EO), which are extracted from the grapefruit peel, are terpenes and terpene oxides [29,30,31]. G-EO is a valuable ingredient because of its characteristic flavor and fragrance [32,33]. G-EO exhibits various biological activities, including free radical scavenging [29,34,35,36,37] and anti-inflammatory [38], anti-bacterial [39,40,41,42], anti-microbial [43], anti-proliferative [37], and anti-cancer activities [44,45,46], and is a positive regulator of sympathetic nervous system activity [47]. The main component of citrus EO is limonene, an important anti-oxidant [48] and anti-inflammatory agent [49]. Inflammatory cell damage and reactive oxygen species (ROS) biosynthesis are inhibited by G-EO treatment through the downregulated expression of IL-1 and COX-2 in *Staphylococcus aureus* (*S. aureus)*-exposed HaCaT keratinocytes [50]. These results indicate that G-EO exhibits a remarkable protective effect in HaCaT keratinocytes by inhibiting inflammatory-stimulus-mediated ROS production. In addition, skin-barrier-structure-associated proteins, such as filaggrin (FLG) and loricrin, are found to be recovered by treatment with G-EO in an *S. aureus*-degraded 3D skin model [50].

### 2.5. Oregano Essential Oil (O-EO)

*Origanum vulgare* (*O. vulgare*), also known as oregano, is a ubiquitous aromatic plant of the Lamiaceae family and a typical Mediterranean flora [51]. Assuming no toxicity, *O. vulgare* is used not only in therapeutic regimens [52,53,54] but also in the food, agricultural, and veterinary fields [55,56]. Moreover, oregano EOs (O-EOs) exhibit a unique function in the prevention of neurodegenerative disorders [57]. The major components of O-EO are carvacrol (CRV) and thymol [51], which exhibit anti-oxidant, immunomodulatory, anti-cancerous, anti-melanogenesis, anti-inflammatory, and anti-microbial activities [52,54,58]. The anti-cancer effects of the EOs, such as their effects against melanoma, are discussed in Section 3. O-EO is found to reduce ROS production, DNA damage, and the expression of inflammatory factors, such as inter-cellular adhesion molecule (ICAM)-1, iNOS, and COX-2, in IFN-γ/histamine-induced NCTC254 cells, a normal human keratinocyte cell line [51]. In addition, the expression levels of extracellular matrix (ECM) agents—such as matrix metalloproteinase (MMP)-1 and MMP-12—and cell proliferation are found to be reduced by O-EO treatment in IFN-γ/histamine-induced NCTC254 cells [51].

### 2.6. Satureja sahendica Essential Oil (SS-EO)

*Satureja sahendica* (*S. sahendica*) is a perennial plant and belongs to the Lamiaceae family [59]. The major constituents of *S. sahendica* EO (SS-EO) are CRV, thymol, p-cymene, ß-caryophyllene, linalool, and other terpenoids with anti-bacterial properties [60]. Assuming no toxicity, the *Satureja* genus is known to exhibit anti-oxidant, anti-bacterial, and anti-inflammatory effects in the management of diarrhea and wound healing [61,62,63]. Topical SS-EO treatment is found to reduce the wound area, bacterial count, and cell infiltration in wound-induced mouse [59]. Mechanically, SS-EO enhances the levels of various factors, such as the anti-inflammatory-associated cytokine, IL-10 [64]; proliferation-associated factors, insulin-like growth factor (IGF)-1, fibroblast growth factor (FGF)-2, and chemokine (C-X-C motif) ligand (CXCL)-1 [65,66,67,68]; and angiogenesis-associated factors, vascular endothelial growth factor (VEGF), and transforming growth factor (TGF)-β [65,69], in isolated primary skin tissues from mice [59]. In addition, SS-EO is also found to downregulate the level of IL-1β in mouse skin tissues [59].

### 2.7. Matricaria chamomilla Essential Oil (MC-EO)

*Matricaria chamomilla* L., also known as German chamomile, is a member of the Compositae family [70]. *Matricaria chamomilla* (*M. chamomilla*) is reported as possessing traditional uses; it is used in the treatment of gastrointestinal conditions [71] and has anti-inflammatory [72] and anti-spasmodic properties [73]. In addition, it is reported that *M. chamomilla* EO (MC-EO) promotes wound healing and repairs the wounded skin barrier [74]. Moreover, Wang et al. found that MC-EO decreased the pro-inflammatory factors such as TNF-α and IL-6 during eczema [75]. The major component of MC-EO is azulene [70]. The inflammatory cytokines—IL-1β, IL-6, and TGF-β—are attenuated by via MC-EO treatment through downregulation of phosphorylated (p)-Akt, the p-mammalian target of rapamycin (p-mTOR), and the p-p38 pathway in IL-22, TNF-α, or LPS-stimulated HaCaT cells [70]. In addition, not only the clinical symptoms of psoriasis, such as erythema, thickening, and scaling, but also the skin inflammation cytokines are alleviated through the downregulation of p-phosphoinositide 3-kinase (PI3K) and p-mTOR by treatment with MC-EO in a mouse model of IMQ-induced psoriasis [70]. Moreover, treatment with MC-EO decreases scratch frequency and serum levels of immunoglobulin (Ig)E, IgG, and histamine in a mouse model of 2,4-dinitrochlorobenzene (DNCB)-stimulated atopic dermatitis (AD) [76]. Moreover, the symptoms of eczema, such as skin erythema, exudation, thickening, rough surface, and swelling, are reduced by treatment with MC-EO in a DNCB-induced eczema mouse model [75]. The serum levels of IL-6, IL-17, and TNF-α are also downregulated through the downregulation of mitogen-activated protein kinase (MAPK) and the NF-κB pathway by treatment with MC-EO in a DNCB-induced eczema mouse model [75].

### 2.8. Helianthus annuus Plant Oil (HA-PO)

*Helianthus annuus* (*H. annuus*), also known as common sunflower, belongs to the Asteraceae family [77]. The major components of *H. annuus* plant oil (HA-PO) are oleic and linoleic acids [77]. The HA-EO moisturizes and protects damaged skin barriers. In particular, ozonated HA-PO is an active-components mixture obtained from the partial ozonation of HA-PO. Ozonated HA-PO possesses an anti-inflammatory effect on the skin diseases of mice and humans [78]. The symptoms of AD, such as scaling, excoriation, erythema, edema, epidermal thickness, infiltration of mast cell, serum levels of IgE, and spleen weight and lymph node length are decreased by treatment with ozonated HA-PO in an oxazolone (Oxz)-induced AD mouse model [79]. In addition, skin hydration function and FLG are recovered by treatment with ozonated HA-PO through the downregulation of IL-4/signal transducers and activators of the transcription (STAT) 3/extracellular signal-regulated kinase (ERK) pathway in an Oxz-induced AD mouse model [79]. Moreover, the levels of nitrite oxide (NO) and iNOS are inhibited by treatment with ozonated HA-PO through downregulation of IL-1β and TNF-α via MAPK and the NF-κB pathway in serum and skin tissues in an Oxz-induced AD mouse model [79].

### 2.9. Mentha arvensis Essential Oil (MA-EO)

*Mentha arvensis* (*M. arvensis*), commonly known as mint, belongs to Lamiaceae family and is a flowering plant species [80]. *Mentha* species are native to Asia, Europe, Africa, Australia, and North America [81]. *M. arvensis* has been commonly used in many medicines for its anti-inflammatory and anti-oxidant activities [82]. The major components of *M. arvensis* EO (MA-EO) are menthol, menthone, and piperitone [80]. The inflammation mediators, such as PGE2 and NO, IL-1β, and IL-6, are decreased by treatment with MA-EO through the downregulation of iNOS and COX-2 in LPS-exposed HaCaT keratinocytes [80]. In addition, the clinical symptoms of AD, such as erythema, edema, and ear thickness, as well as the scoring AD index, are improved by treatment with MA-EO in a DNCB-induced AD mouse model [80]. Moreover, infiltration of mast cell and epidermal layer thickness are attenuated by treatment with MA-EO in a DNCB-induced AD mouse model [80]. Furthermore, the formation of the nucleotide-binding oligomerization-domain-like receptor (NLR) family pyrin-domain-containing 3 (NLRP3) inflammasome is attenuated by treatment with MA-EO in the macrophage of a DNCB-induced AD mouse [83].

### 2.10. Rosmarinus officinalis Essential Oil (RO-EO)

*Rosmarinus officinalis* (*R. officinalis*) L., also known as rosemary, is a perennial evergreen shrub [84]. Rosemary is often used to treat digestive problems, the nervous system, and allergies in Morocco [85]. Takaki et al. assessed the anti-inflammatory effect of *R. officinalis* EO (RO-EO) and reported that RO-EO treatment reduced effusion volume and leukocyte migration in a carrageenan-stimulated rat model [86]. The major components of RO-EO are camphor and eucalyptol [84]. The symptoms of AD are improved by treatment with RO-EO in a DNCB-induced AD mouse model [84]. In addition, serum levels of IL-6 and TNF-α are attenuated by treatment with RO-EO through regulation of the Janus kinase (JAK)/STAT/MAPK pathway in a DNCB-induced AD mouse model [84].

### 2.11. Curcuma longa Essential Oil (Cl-EO)

*Curcuma longa* (*C. longa*), is a member of the Zingiberaceae family and is grown mainly in Asia and India [87]. The skin penetration effects of the rhizome extracts, molecules, and EO of *Curcuma* species in skin diseases are reported, assuming no toxicity [88]. The major components of *C. longa* EO (Cl-EO) are terpinolene and α-phellandrene [89]. The levels of inflammatory cytokines, including, IL-6, IL-1β, and TNF-α, are downregulated by treatment with Cl-EO in LPS- or TPA-stimulated HaCaT cells [89]. In in vivo systems, the levels of IL-1β, IL-6, and TNF-α are decreased by treatment with Cl-EO in the serum of a TPA-induced inflammatory mouse model [89]. In addition, ear edema and leucocyte infiltration are reduced by treatment with Cl-EO in a TPA-induced inflammatory mouse model [89].

### 2.12. Artemisia argyi Essential Oil (AA-EO)

*Artemisia argyi* (*A. argyi*) is a species of herbaceous plant distributed in China, Japan, and many parts of Taiwan [90,91]. *A. argyi* has been determined to possess various biological properties, including an anti-mutagenic [92], anti-cancer [93,94,95,96], anti-inflammatory [97], and anti-oxidant [91] properties. Additionally, the biological properties of *A. argyi* EO (AA-EO) have been studied, such as its anti-asthmatic [98] and anti-fungal activity [99]. The major components of AA-EO are cineole, camphor, eucalyptol, and caryophyllene [90,91]. Ear edema, as well as neutrophil infiltration, hyperplasia of the epidermal layer, and disruption of connective tissue, are reduced by treatment with AA-EO through downregulation of the JAK/STAT/COX-2 pathway in a TPA-induced inflammatory mouse model [90].

### 2.13. Lavender-Essential Oil (L-EO)

*Lavandula angustifolia*, also known as lavender, belongs to the Labiatae family and has been used as either a form of dried plant or for its volatile oils due to its diverse therapeutic and cosmetic properties [100]. Lavender EO (L-EO) has been reported to possess numerous biological properties, including anxiolytic [101], neuroprotective [102], anti-oxidant [103], analgesic [103], anti-inflammatory [103,104], anti-microbial [105,106], wound healing [107], and anti-joint-pain properties [108]. The symptoms of psoriasis, such as thickness, erythema, and scaling, and the inflammatory cytokines levels of T-helper (Th)-17-specific cells, such as IL-17 and IL-22, and Th-1-specific cells, such as TNF-α and IL-1β, are reduced by treatment with L-EO in an IMQ-induced psoriasis mouse model [109]. In addition, the major compounds of L-EO are linalool and linalyl acetate [107], which represent the alleviation effects of psoriasis, such as the reduction of erythema, thickness, scaling, keratin, pigmentation, and curvature, as well as the inflammatory cytokines levels of Th-17- and Th-1- specific cells by downregulating C-C motif chemokine receptor (CCR) 6 and IL-17 expression in an IMQ-induced psoriasis mouse model [109]. In addition, linalool and linalyl acetate exhibit an anti-inflammatory effect against a carrageenan-induced edema model [110].

### 2.14. Zanthoxylum coreanum Essential Oil (ZC-EO)

The *Zanthoxylum* species belongs to the Rutaceae family and has been used as a source of spices in Asian cuisine and traditional Asian medicine [111,112]. *Zanthoxylum coreanum* (*Z. coreanum*) has been shown to have anti-viral activity against picornaviruses [113]. The major components of *Z. coreanum* EO (ZC-EO) are β-Ocimene and α-pinene [114]. The AD-like skin lesions, such as large ulcers, ear swelling, and hyperkeratosis, the thickness of the epidermis and dermis, and inflammatory cell infiltration, are inhibited by treatment with ZC-EO through the downregulation of NF-κB and the phosphorylated MAPK pathway in a DNCB-induced AD mouse model [114].

## 3. Anti-Cancer Effect of EOs

EOs are concentrated hydrophobic liquids from aromatic plants that exert anti-cancer effects on various cellular targets [115]. Melanogenesis is a pivotal process in melanocytes, which possess melanosomes to synthesize and store melanin pigment [116]. This process is tightly regulated by several enzymes, including tyrosinase and tyrosinase-related proteins (TRPs)-1 and -2 [117]. These enzymes are responsible for initiating and regulating melanogenesis [117]. In addition, these enzymes contribute to the completion of the process and act as modifiers to regulate pathway velocity [118]. Among melanogenesis-associated enzymes, TRP-1 and TRP-2 stabilize tyrosinase activity and maintain the structural integrity of melanosome [118]. Moreover, melanogenesis is associated with H_2_O_2_ production through the enzymatic and non-enzymatic reactions and subsequently induces oxidative stress in melanocytes [119,120]. ROS production is derived by α-melanocyte, stimulating hormone (MSH)-induced melanogenesis [121]. Treatment of several ROS scavengers and ROS inhibitors reduces the UV-induced melanogenesis [122,123]. Therefore, the development of melanogenesis inhibitors, anti-oxidants, and ROS scavengers has been revealed to be beneficial in the treatment of hyperpigmentation in skin care fields. This section is focused on EO’s anti-cancer effects, including their anti-melanoma effects.

### 3.1. Aloysia citrodora Essential Oil (AC-EO)

**Figure 2:**
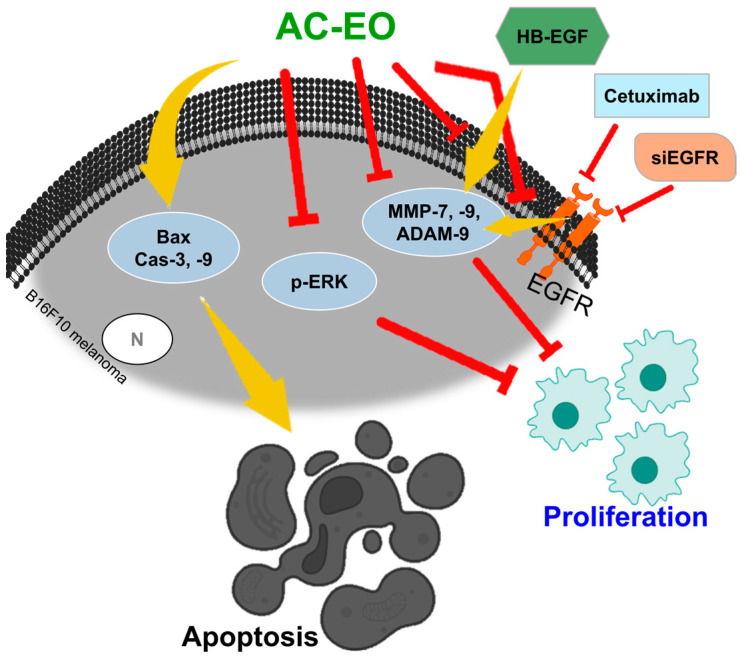
Schematic of the anti-melanoma effect of AC-EO treatment on B16F10 melanoma cells. Cell viability is regulated by AC-EO-mediated apoptosis or the downregulation of proliferation [133]. HB-EGF or EGFR overexpression affects MMPs-7 and -9 and ADAM-9 signaling, whereas AC-EO, Cetuximab, or siRNA-EGFR inhibits signaling [133]. AC-EO: *Aloysia citrodora*-essential oil; Bax: Bcl-2-associated X; Cas: caspase; p-ERK: phosphoextracellular signal-regulated kinase; MMP: matrix metalloproteinase; ADAM: a disintegrin and metalloprotease; HB-EGF: heparin-binding epidermal growth factor; EGFR: epidermal growth factor receptor.

*Aloysia citrodora* (*A. citrodora*) Paláu (Lippia citriodora Kunth), also known as Aloysia. triphylla (L’Hér.) Britton, Aloysia. citridora, Aloysia. citriodora Paláu, Lippia citriodora Kunth, Lippia. citrodora Kunth, Lippia. triphylla (L’Hér.) Kuntze, Lippia triphylla, Verbena triphylla, Aloysia triphylla, Verbena citriodora, or lemon verbena, is derived from America, Africa, and Europe, and is used in pharmacological purposes to alleviate flatulence, diarrhea, rheumatoid arthritis, and insomnia [124,125]. The A. citrodora EO (AC-EO) contains the chemical metabolites of aromatic plants, such as neral, geranial, limonene, citral, and 1,8-cineole [124,126]. AC-EO and its components (geranial, neral, flavonoid, and phenol) have been reported to possess anti-cancer effects in breast cancer, human chronic myelogenous erythroleukemia, malignant tumor cell lines, a 4T1 breast cancer xenograft mouse model, murine mastocytoma cell line, colorectal adenocarcinoma, hepatocellular carcinoma, breast adenocarcinoma, and malignant melanoma [127,128,129,130]; anti-oxidant effects [131,132]; and anti-bacterial effects [131]. In the presence of AC-EO, cell viability is dose-dependently decreased through the downregulation of p-ERK expression and the upregulation of apoptotic factors such as Bax and caspases-3 and -9 in human epidermoid carcinoma A431, human SK-MEL-28 melanoma, and murine B16F10 melanoma [133]. In addition, the tumor volume is found to be reduced by treatment with AC-EO in a melanoma mouse model [133]. ECM-degrading proteases, including disintegrin and metalloprotease-9 (ADAM-9), MMP-2, -7, and -9, play pivotal roles in cancer processes, including the growth, migration, invasion, adhesion, proliferation, and apoptosis of melanoma [134,135,136,137,138,139]. ECM degradation and MMP expression are modulated by epidermal growth factor receptor (EGFR) signaling [140,141]. The plasma and expression levels of MMP-7 and -9 and ADAM-9 are reduced by AC-EO treatment in B16F10-injected mice and in B16F10 melanoma [133]. EGFR overexpression or heparin-binding (HB)-EGF treatment-mediated cell proliferation and MMP-7, -9, and ADAM-9 expression levels are also found to be reduced by treatment with AC-EO [133]. The recombinant chimeric EGFR monoclonal antibody, cetuximab, induces EGFR silencing [133]. Furthermore, co-treatment with AC-EO and siRNA-EGFR or cetuximab leads to a more effective downregulation of cell proliferation and the expression level of MMP-7 and -9 than individual treatment in B16F10 melanoma [133]. The anti-melanoma effects of AC-EO are shown in Figure 2. Overall, AC-EO is involved in the inhibition of EGFR signaling and may be a therapeutic agent against melanoma.

More effective carriers are available for maintaining the solubility of EOs. Chitosan nanoparticles, which possess biodegradability, biocompatibility, and mucosal adhesion properties, are among the most-common carriers [142,143]. Nanoparticle-based structures containing EOs are promising for improving the solubility and efficacy of EOs [144,145]. DPPH radical-scavenging activity is found to be enhanced by treatment with chitosan nanoparticles containing citral and *Lippia citriodora* EO (LC-EO) [126]. Several researchers have reported the anti-cancer effects of chitosan nanoparticles containing EOs, such as *Syzygium aromaticum* EO in A375 melanoma cells [146], *Morinda citrifolia* EO in A549 cells [147], and celandine (*Chelidonium majus* L.) EO in MCF-7 cells [148]. Cell viability has been found to decrease in a dose-dependent manner following chitosan nanoparticle-conjugated citral or LC-EO treatment in A375 cells [126].

### 3.2. Origanum majorana Essential Oil (OM-EO)

The major constituents of *Origanum majorana (O. majorana)* EO (OM-EO) are Terpinen-4-o1 and L-α-Terpineol [149]. OM-EO exhibits anti-bacterial, anti-oxidant, and anti-fungal effects [150]. Nanoemulsions are dispersions of oil in water or water in oil, formulated using amphiphilic materials (surfactants), where the droplets are on the nanometer scale [151]. Nanoemulsions have important advantages, including high stability, bioavailability, biocompatibility, and biodegradability [149], and are among the most suitable formulations with which to enhance the efficiency and stability of EOs [152]. Recently, several studies have reported the anti-cancer effects of nanoemulsions containing EOs, such as *Cinnamomum cassia* EO, *Zingiber ottensii* EO, and *Citrus aurantium* in A549 [153,154,155]. Notably, treatment with nanoemulsions containing OM-EO enhances DPPH radical-scavenging activity and apoptotic effects in A375 melanoma cells [149].

### 3.3. Artemisia capillaris Grass Clumps Essential Oil (AC-EO)

The ethanol extract and ethyl acetate fraction of *Artemisia capillaris* (*A. capillaris*) possess anti-cancer [156] and anti-oxidant functions [157], respectively. Treatment with *A*. *capillaris* grass clumps EO (AC-EO) is found to reduce both intracellular and extracellular melanin contents via the downregulation of TRP-1 signaling in melanoma B16F10 melanoma [118]. The skin is exposed to various external stresses, such as ROS, which induce various deleterious effects and the apoptosis of keratinocytes [158,159]. AC-EO treatment is found to upregulate the proliferation of H_2_O_2_-exposed B16F10 melanoma [118].

### 3.4. Camellia japonica Seed Essential Oil (CJ-EO)

*Camellia japonica* (*C. japonica*) seed EO (CJ-EO) possesses various biological activities, including anti-oxidant [160,161], anti-bacterial [162,163], anti-inflammatory [164], and skin barrier function [165]. Tyrosinase is a critical enzyme for melanin synthesis [166]. Therefore, inhibition of substances of tyrosinase, TRP-1, and TRP-2 reduces melanin synthesis [167]. More recently, the major components of CJ-EO are identified as hexamethylcyclotrisiloxane and octamethylcyclotetrasiloxane [168]. The activities of melanin synthesis-associated components, tyrosinase, and TRP-1 and -2, as well as the content of melanin, are dose-dependently reduced by CJ-EO treatment in α-MSH-exposed B16F10 melanoma [168]. Thus, CJ-EO could be considered as a potential whitening agent.

### 3.5. Origanum syriacum (OS) and Origanum Ehrenbergii (OE)

*Origanum syriacum* (OS) and *Origanum ehrenbergii* (OE) are two naturally growing plants in Lebanon that belong to the Lamiaceae family [57]. These plants are used in maceration for rheumatism and neuralgic treatments [169]. The major components of OS and OE are aromatic terpenoids, quinones, and CRV [170]. The cell viability of B16-F1 melanoma is found to be dose-dependently decreased following treatment with OS-EO and OE-EO [171]. Melanin levels are downregulated by treatment with OS-EO, OE-EO, or CRV without any alterations to tyrosinase activity in B16-F1 melanoma [171]. These results indicate that both EOs and CRV exhibit anti-melanogenic activity by competing with tyrosine as a tyrosinase substance.

### 3.6. Calocedrus formosana Essential Oil (CF-EO)

*Calocedrus formosana (C. formosana)*, also known as Taiwan incense cedar, belongs to the Cupressaceae family [172]. The compounds of *C. formosana* possess the diverse biological effects, such as anti-cancer [173], anti-oxidative [174,175], anti-inflammation [176], and anti-fungal [177] effects. The major components of *C. formosana* EO (CF-EO) are α-Terpineol, Terpinen-4-o1, and thymol [172]. The treatment with CF-EO inhibits melanogenesis by inhibiting tyrosinase activity and by reducing TRP-1 and TRP-2, co-stimulated with α-MSH and forskolin (FSK), in B16F10 melanoma [172]. Furthermore, thymol inhibits melanogenesis in the presence of co-stimulation with α-MSH and FSK in B16F10 melanoma [172].

### 3.7. Melaleuca quinquenervia Essential Oil (MQ-EO)

*Melaleuca quinquenervia* (*M. quinquenervia*) belongs to the Myrtaceae family and is native to northern Australia [178]. The major compositions of *M. quinquenervia* EO (MQ-EO) are the monoterpene family, such as 1,8-cineole, α-pinene, and α-terpineol [179]. Among them, 1,8-cineole is responsible for the anti-bacterial activity of MQ-EO [180]. The melanin production is inhibited by treatment with MQ-EO but also its major compositions, 1,8-cineole, α-pinene, and α-terpineol, through the downregulation of tyrosinase activity in α-MSH-exposed B16 melanoma [179]. It is well known that α-MSH-exposed melanogenesis is involved in ROS production [121,181]. Oxidative damage and lipid peroxidation is recovered by treatment with MQ-EO through the upregulation of glutathione (GSH) levels or anti-oxidants, such as superoxide dismutase (SOD), glutathione peroxidase (GPx), and catalase (CAT), in α-MSH-stimulated B16 melanoma [179]. GSH plays vital role in anti-melanogenesis and in the maintenance of cellular redox status [182]. The anti-melanogenesis effect of MQ-EO might be regulated by the regulation of tyrosinase activity and anti-oxidant ability.

### 3.8. Cinnamomum cassia Essential Oil (CC-EO)

*Cinnamomum cassia* (*C. cassia*) Presl is broadly grown in China [167]. Assuming no toxicity, the dried form of *C. cassia* stem bark is known to exhibit multiple biological activities, such as anti-bacterial [183], anti-inflammatory [184], and anti-diabetic properties [185], and its traditional uses are reviewed in [186]. Additionally, it has been found that the extraction of *C. cassia* twigs suppresses tyrosinase activity [187]. The *C. cassia* EO (CC-EO) is known to possess hypouricemic [188] and anti-fungal activities [189]. The major components of CC-EO are cis-2-methoxycinnamic acid and cinnamaldehyde [167]. Melanin content is reduced by treatment with CC-EO or its major components through the inhibition of tyrosinase activity and its expression in α-MSH-induced B16 melanoma [167]. α-MSH-mediated oxidative stress and lipid peroxidation are attenuated by treatment with CC-EO or cinnamaldehyde through the regulation of GSH level and CAT activity in α-MSH-induced B16 melanoma [167].

### 3.9. Leaf of Alpinia nantoensis Essential Oil (LAN-EO) and Rhizome of Alpinia nantoensis Essential Oil (RAN-EO)

*Alpinia* is the largest genus in Zingiberaceae, which are found in tropical/sub-tropical regions of China, India, and Polynesia [190]. Extractions from the various parts of *Alpinia nantoensis* (*A. nantoensis*), such as the leaf, stem, and rhizome, exhibit anti-metastatic properties in lung cancer [191] and breast cancer cells [192]. Treatment with the leaf of *A. nantoensis* EO (LAN-EO) or rhizome of *A. nantoensis* EO (RAN-EO) reduces melanogenesis through ERK1/2-activation-mediated MITF ubiquitination and proteasomal degradation, which downregulate tyrosinase and TRP-1 in FSK-stimulated B16F10 cells [193]. The major compositions of LAN-EO and RAN-EO are camphor, camphene, β-pinene, p-cymene, α-pinene, and D-limonene [193]. Among them, α-pinene and D-limonene attenuate melanogenesis by downregulating tyrosinase activity in FSK-stimulated B16F10 melanoma [193].

### 3.10. Chrysanthemum boreale MAKINO Essential Oil (CB-EO)

*Chrysanthemum boreale* (*C. boreale*) MAKINO is a perennial herbaceous plant and is a member of the Compositae family [194]. The *C. boreale* species, including *C. boreale* MAKINO, are broadly grown in East Asia, including Korea, China, and Japan [195,196], and, assuming no toxicity, they are widely used as medicinal plants due to their biological properties, such as anti-inflammation and anti-bacterial action, skin-regenerative properties, and wound healing properties [194,196,197]. The major compounds of CB-EO are camphor, germacrene-D, and β-thujone [196]. Moreover, treatment with *C. boreale* MAKINO EO (CB-EO) from the flower reduces melanogenesis through the downregulation of tyrosinase via the ERK and MAPK pathway in α-MSH-stimulated B16BL6 melanoma [198].

### 3.11. Vitex negundo Essential Oil (VN-EO)

*Vitex negundo* (*V. negundo*) Linn is a large aromatic shrub belonging to the Verbenaceae family, mainly distributed in the Indochina region [199]. *V. negundo* has been revealed to possess various pharmacological properties, including anti-nociceptive [200,201], anti-convulsant [202], and anti-inflammatory [201] properties. In addition, the extracts of *V. negundo* Linn are known to possess analgesic [203], anti-inflammatory [203], and tyrosinase inhibition properties [204]. The major components of *V. negundo* EO (VN-EO) are sesquiterpene and monoterpene [199]. Treatment with VN-EO reduces melanogenesis through the downregulation of tyrosinase activity in α-MSH-induced B16F10 cells [199]. Moreover, the anti-oxidant effects of VN-EO are revealed through its DPPH radical-scavenging activity [199].

### 3.12. Achillea millefolium Essential Oil (AM-EO)

The *Achillea* genus of the Asteraceae family is broadly distributed throughout the world [205], and among this genus is *Achillea millefolium* (*A. millefolium*) L., which is used to treat gastrointestinal disorders [206], hepatobiliary conditions [207], and cardiovascular [208] and respiratory infections [209]. The major components of *A. millefolium* EO (AM-EO) are camphor, artemisia ketone, linalyl acetate, and 1,8-cineole [210]. Among them, linalyl acetate inhibits melanin contents by downregulating tyrosinase activity in α-MSH-induced B16 melanoma [211]. Treatment with AM-EO reduces melanogenesis through the regulation of tyrosinase activity and the Jun N-terminal kinase -ERK pathway in α-MSH-stimulated B16 melanoma cells [211]. Moreover, oxidative stress and lipid peroxidation are attenuated by treatment with AM-EO through the regulation of the GSH level and anti-oxidant enzymes in α-MSH-stimulated B16 cells [211].

### 3.13. Artemisia argyi Essential Oil (AA-EO)

The anti-inflammatory activity of AA-EO has been addressed in Section 2.12. Melanogenesis is attenuated by treatment with AA-EO through the downregulation of tyrosinase activity or oxidative stress in α-MSH-stimulated B16F10 cells [91].

### 3.14. Vetiveria zizanioides Essential Oil (VZ-EO)

*Vetiveria zizanioides* (*V. zizanioides*) is a perennial tussock grass that belongs to of the Gramineae family [212,213]. Moreover, the various parts or volatile oils of *V. zizanioides* possesses diverse biological properties, including anti-oxidant [214], anti-microbial [215], and anti-inflammatory properties [216]. The major components of *V. zizanioides* EO (VZ-EO) are Cedr-8-en-13-ol and α-Amorphene [217]. The melanin production is attenuated by treatment with VZ-EO through the inhibition of tyrosinase activity and anti-oxidant activity via the regulation of GSH level and anti-oxidant enzymes, GPx and SOD, in α-MSH-stimulated B16 melanoma cells [217].

### 3.15. Eucalyptus camaldulensis Essential Oil (EC-EO)

Eucalypt trees belong to the *Eucalyptus* genus and Myrtaceae family [218]. The *Eucalyptus* genus is derived from Australia [219]. The diverse pharmacological activities of *Eucalyptus* species leaf EO, such as its anti-bacterial [220], anti-termitic [221], and anti-oxidant activities [222], have been reported. The major components of *Eucalyptus camaldulensis* EO (EC-EO) are eucalyptol and γ-terpinene [223]. Melanogenesis is reduced by treatment with EC-EO through the downregulation of MAPK/PKA signaling pathways and TRP-1 activities in α-MSH-stimulated B16F10 cells [223].

### 3.16. Acorus macrospadiceus Essential Oil (AM-EO)

Plants of the *Acorus* species belong to the Acoraceae family and are distributed in India, Europe, and Asia [224]. Assuming non-toxicity, the biological activities of the EOs extracted from *Acorus* species are reported. The volatile oils from *Acorus (A.) calamus* rhizomes possess diverse biological properties, including anthelmintic activities [225] and acetylcholinesterase-inhibitory activities [226]. In addition to *Acorus calamus*, the volatile oil from *A. gramineus* rhizomes possesses a neuroprotection function [227]. The major components of *A. macrospadiceus* EO (AM-EO) are chavicol methyl ether and nootkatone [228]. Melanogenesis is reduced by treatment with AM-EO through the downregulation of tyrosinase activity or oxidative stress in α-MSH-stimulated B16F10 cells [228].

## 4. Modulation of Skin Proliferation

Wound healing is complex physiological process which consists of three dynamic steps, including an inflammatory step, a proliferative step, and a maturation step involving the development of cellular connective tissue and the formation of newly generated epithelial tissue [229,230]. The cellular connective tissue is supported by the presence of collagen [231]. In addition, dysregulated keratinocyte growth and differentiation lead to aberrant cellular processes, including hyperproliferation, abnormal differentiation, and inflammatory infiltration [232]. The hyperproliferation of epidermal keratinocytes leads to the pathogenesis of several cutaneous disorders, such as psoriasis and AD [233]. These diseases are characterized by dysregulated epidermal homeostasis, which causes disordered skin proliferation and differentiation [234]. This section is focused on the effects of EOs on skin proliferation and regeneration in maintaining epidermal homeostasis.

### 4.1. Artemisia montana Pampan Essential Oil (AM-EO)

The *Artemisia* genus has been used to treat cancer, malaria, inflammation, and virus infection [235,236,237]. In particular, *Artemisia montana* (*A. montana*) Pampan engages in various biological activities, including anti-diabetic [238], anti-inflammatory [239], and anti-oxidant activities [240]. The major components of *A. montana* EO (AM-EO) are β-caryophyllene, germacrene D, 1,8-cineole, and camphor [241]. AM-EO treatment is found to enhance the levels of p-Akt and p-ERK1/2 in HaCaT keratinocytes [241]. In addition, type-IV collagen synthesis promotes basement membrane formation in the skin [242]. Type-IV collagen synthesis is upregulated by *AM-EO* treatment in HaCaT keratinocytes [241]. AM-EO treatment increases wound closure in the rat tail [241], suggesting that AM-EO reveals a potential positive effect on skin regeneration.

### 4.2. Pistacia lentiscus L. Plant Oil (PL-PO)

*Pistacia lentiscus* L., which is known as the lentisk or mastic tree in Greece, belongs to the Anacardiaceae family [243]. *Pistacia lentiscus* PO (PL-PO) is mainly used as a flavor agent in cuisines but is also administered as an ointment to treat wounds [244]. The major components of PL-PO are oleic, palmitic, linoleic acids, and phenolic compounds, including tocopherols, carotenoids, and anthocyanins [245,246]. PL-PO possesses several biological activities, including anti-bacterial, anti-oxidant, proliferation, and wound healing effects [247,248,249,250]. Treatment with PL-PO liposomes induces the proliferation of HaCaT keratinocytes [251] and enhances the migration of HaCaT keratinocytes [251]. The application of liposomes with PL-PO might be a useful approach in the field of cosmetics.

### 4.3. Lavender-Essential Oil (L-EO)

L-EO is derived from the blossoms of *Lavandula angustifolia* [252]. As described in Section 2.13, the major components of L-EO are linalool and linalyl acetate [107]. Based on various reports, L-EO is expected to exhibit beneficial effects in wound healing [253,254,255,256]. Wound area is found to be rapidly reduced by treatment with L-EO compared with that of the control in a rat model [107]. The synthesis of type-III collagen (Col III) a1, an essential component for the formation of granulation tissue in the early phase of wound healing, is upregulated by L-EO treatment in rat wound lesions [107]. In addition, the mRNA levels of Col IIIa1 and Col Ia2 are enhanced by L-EO treatment in a wounded rat model [107]. TGF-β is known to induce the fibroblasts proliferation and production of both Col I and Col III [257]. The level of TGF-β is upregulated by L-EO treatment in the wound lesions of rats [107]. In addition, TGF-β induces the differentiation of fibroblasts to myofibroblasts in wound granulation tissue [258]. Differentiation into myofibroblasts and wound contraction are observed in rat wound lesions treated with L-EO [107].

### 4.4. Calophyllum inophyllum Plant Oil (CI-PO)

*Calophyllum inophyllum* (*C*. *inophyllum*), a member of the mangosteen family, is a large, evergreen tree of South India, Malaysia, Africa, Polynesia, and the Philippines [259]. *C. inophyllum* PO (CI-PO; syn: Tamanu oil) is a plant oil acquired from the seeds of the *C. inophyllum* tree [260]. CI-PO possesses anti-inflammatory [261], anti-microbial [262,263], and anti-fungal [263]. The components of *C. inophyllum* are calophyllolide, calophyllic acid, inophyllum, and polyphenols that possess wound healing and anti-oxidant properties [264]. Wound area is reduced by treatment with CI-PO through the enhancement of mature granulation and the density of fibrosis and collagen in wounded skin in a rat model [265].

### 4.5. Parrotiopsis jacquemontiana Plant Oil (PJ-PO)

*Parrotiopsis jacquemontiana* (*P. jacquemontiana*) belongs to the Hamamelidaceae family [266] and is reported to possess medicinal properties such as anti-microbial [266] and anti-cancer properties [267]. The major compounds of *P. jacquemontiana* PO (PJ-PO) are 2, 6-dimethyl-8-oxoocta-2, 6-dienoic acid, syringol, and catechol [268]. Wound contraction is inhibited by treatment with PJ-PO through collagen synthesis in wounded skin in a rat model [268]. In addition, the thickness of the epidermis layer is accompanied by faster fibroblast cells migration from the dermal layer to the epidermal layer in the treated groups [268].

### 4.6. Chrysanthemum boreale Makino Essential Oil (CB-EO)

The anti-melanogenesis activity of CB-EO has been addressed in Section 3.10. The keratinocyte proliferation is enhanced by treatment with CB-EO through the upregulated Akt and ERK1/2 pathway in HaCaT keratinocyte [196]. In addition to the in vitro model, wound area is decreased by treatment with CB-EO in a wounded tail in a rat model [196].

### 4.7. Salvia aurea L. Essential Oil (SA-EO)

*Salvia aurea* L. (syn. *S. africana-lutea* L.) belongs to the Lamiaceae family [269], and, assuming non-toxicity, *Salvia* L. genus represents various pharmacological activities, including anti-fungal [270], anti-bacterial [271], anti-inflammatory [272], anti-cancer [273], and anti-oxidant activities [271]. In addition, the *Salvia* species’ essential oil is reported to possess anti-fungal [274] and anti-viral activities [275]. The major compounds of *Salvia aurea* L. EO (SA-EO) are 1,8-cineole, β-pinene, cis-thujone, and camphor [276]. Wound closure is increased by treatment with SA-EO in NIH 3T3 fibroblasts [276].

### 4.8. Rose Plant Oil

*Rosa damascena* Mill. F. trigintipetala Dieck (Rosaceae) is a plant rich in polyphenolic compounds with various pharmacological properties, including anti-virus, anti-bacterial, anti-oxidant, anti-tussive, anti-diabetic, anti-plasmodial, and anti-inflammatory properties [277]. Polyphenols, especially flavonoids, are well known to possess a broad range of biological activities, such as anti-oxidant, anti-cancer, anti-inflammatory, anti-mutagenic, and anti-proliferative [278]. Quercetin and ellagic acid, which are polyphenols and flavonoids enriched in *Carya illinoinensis* (Wangenh.) K. Koch, *Juglans nigra* L., *Rosa rugosa* Thunb., *Prunus domestica* (Rosaceae), *Malus domestica* (Rosaceae), and *Prunus avium* (Rosaceae), are found to exert anti-proliferative effects on keratinocytes, liver cancer, and breast cancer [232,279,280,281,282,283,284]. In addition, the combined administration of quercetin and ellagic acid induces synergistic effects on apoptosis and anti-proliferation in MOLT-4 human leukemia cells [285]. Thus, treatment with rose plant oil distillation wastewater (RODW) decreases the proliferation and migration of HaCaT keratinocytes [232]. Further, the secretion of the proliferation factor, VEGF, is inhibited by RODW treatment in TNF-α-exposed HaCaT keratinocytes [232]. Apoptotic vesicles are induced by RODW treatment in HaCaT keratinocytes [232]. Thus, RODW might be a useful factor in the alleviation of hyperproliferative skin diseases, such as psoriasis.

### 4.9. Zanthoxylum bungeanum Maxim Essential Oil (ZB-EO)

*Zanthoxylum bungeanum* (*Z. bungeanum*) Maxim is widely distributed in most parts of China and several Southeast Asian countries [286]. *Z*. *bungeanum* Maxim EO (ZB-EO) possesses diverse pharmacological properties, such as anti-oxidant [287], anti-cancer [288], anti-inflammatory [289], anti-microbial [290], and insecticidal properties [291]. The major components of ZB-EO are D-limonene and β-myrcene [286]. Treatment with ZB-EO is found to reduce the viability and proliferation of HaCaT keratinocytes through cell cycle arrest [286]. Moreover, the apoptotic effect of ZB-EO is recognized to be mediated by the regulation of apoptosis-associated factors, such as Bax, Bcl-2, and cleaved caspase-3, -8, and -9 in HaCaT keratinocytes [286]. ZB-EO is also considered a potential anti-proliferative drug against hyperproliferative diseases.

### 4.10. Prunus armeniaca Essential Oil (PA-EO)

*Prunus armeniaca* L. (*P. armeniaca*), also known as bitter apricot, belongs to the Rosaceae family and is native to Eurasia and America [292]. Bitter apricot seeds have been used for the alleviation of several skin diseases, including dandruff, acne vulgaris, and furuncle [293]. In addition, Bitter apricot seed possesses a broad range of biological effects, such as anti-cancer [294], anti-oxidant [295,296], anti-microbial [294,295,297], anti-inflammatory [298], and anti-asthmatic [299] activities. Moreover, EO from the apricot seed is reported to possess anti-microbial activity and induce the apoptosis process through regulation of apoptotic factors Bax and Bcl-2 expression [293,300]. The major compounds of *P. armeniaca* EO (PA-EO) are benzaldehyde, benzoic acid, mandelonitrile [301]. The keratinocyte proliferation is inhibited by treatment with PA-EO or benzaldehyde through upregulated apoptosis-associated factors, such as cleaved Poly(ADP-ribose) polymerase (PARP), caspase-3/8/9, and Bax, and downregulated apoptosis and proliferation-associated factors, such as Bcl-2 and NF-κB, in HaCaT keratinocyte [301].

## 5. Summary

Naturally derived EOs and other plant-based active components are believed to possess various biological pharmacological properties and are used as raw materials in drug development. Owing to the various functions of EOs and their active components in the skin, they are considered to be potential treatments for different diseases (Table 1). Although EOs have various and positive effects not only on the skin but also on other tissues and organs, EOs are associated with several drawbacks, such as hypersensitivity reactions, appropriate doses, individual reaction differences, toxicity, and side effects. Because of the stratified structure, various cell types, and the presence of pigmentation in the skin tissue, research on the modulation of skin homeostasis should be dealt with in particular. Thus, scientific evidence pertaining to EOs, such as the identification of the biological and physiological mechanisms of EOs, should be developed and reinforced for skin safety concerns. Moreover, it is necessary to overcome skin cancer and skin diseases, including psoriasis, eczema, and fungal infections, through the discovery of numerous potential EO substances as alternatives to synthetic drugs.

## Figures and Tables

**Table 1:** Different functions of essential oils in the skin and their natural sources.

Essential Oil (EO)/Plant Oil (PO)	Effects	Sources	Parts (Components)	Refs.
*Citrus limetta* peels EO	Anti-inflammatory and anti-oxidant	*C*. *limetta* Risso	Fruit peels (flavonoids)	[4,5,8,9]
*Baccharis dracunculifolia* EO	Anti-inflammatory, immunomodulator, anti-bacterial, anti-diabetic, and anti-microbial	*B*. *dracunculifolia*	Leaves	[10,11,12,13,14,15,16,17,18]
*Perilla frutescens* L. Britt EO	Anti-inflammatory, anti-bacterial, anti-oxidant, alleviation of cold, cough, nausea, vomiting, abdominal pain, constipation, asthma, and food poisoning	*P*. *frutescens* L. Britt	Stems and leaves	[19,20,21,22]
Grapefruit EO	Anti-inflammatory, anti-microbial, anti-oxidants, anti-bacterial, anti-proliferation, anti-cancer, and muscle sympathetic nerve activity regulator	*C*. *maxima* Burm. Merr	Grapefruit peel (terpenes and terpene oxides)	[29,30,31,34,35,36,37,38,39,40,41,42,43,44,45,46,47,50]
Oregano EO	Anti-inflammatory, prevention of neurodegenerative disorders, anti-oxidant, immunomodulatory, anti-cancer, anti-melanogenesis, and anti-microbial	*O*. *vulgare*	CRV, thymol	[51,52,53,54,57,58]
*Satureja sahendica* EO	Anti-inflammatory, anti-bacterial	*S*. *sahendica*	CRV, thymol, p-cymene, ß-caryophyllene, linalool, and other terpenoids	[59,60,64]
*Matricaria chamomilla* EO	Anti-inflammatory, alleviation of gastrointestinal conditions, anti-spasmodic, and wound healing	*M. chamomilla*	Azulene	[71,72,73,74]
*Helianthus annuus* PO	Anti-inflammatory, skin hydration	*H. annuus*	Oleic acid, linoleic acids	[77,79]
*Mentha arvensis* EO	Anti-inflammatory, anti-oxidant,	*M. arvensis*	Menthol, menthone, and piperitone	[80,82]
*Rosmarinus officinalis* EO	Anti-inflammatory, treat digestive problem, nervous system, and allergy	*R* *. officinalis*	Camphor, eucalyptol	[84,85,86]
*Curcuma longa* EO	Anti-inflammatory, skin penetration	*C. longa*	Rhizome (terpinolene and α-phellandrene)	[87,88,89]
*Artemisia argyi* EO	Anti-inflammatory, anti-mutagenic, anti-tumor, anti-oxidant, anti-asthmatic, anti-fungal, and anti-melanogenesis	*A. argyi*	Leaves (cineole, camphor, eucalyptol, and caryophyllene)	[90,91,92,93,94,95,96,97,98,99]
Lavender EO	Anti-inflammatory, skin protection and wound healing, anxiolytic, neuroprotective, anti-oxidant, analgesic, anti-microbial, and alleviation of joint pain	*L*. *angustifolia*	Blossoms (Linalool and linalyl acetate)	[100,101,102,103,104,105,106,107,108,109,110,252,253,254,255,256]
*Zanthoxylum coreanum* EO	Anti-inflammatory, anti-viral	*Z* *. coreanum*	β-Ocimene, α-pinene	[111,112,113,114]
*Aloysia citrodora* EO (*Lippia citriodora* EO)	Anti-melanoma, anti-cancer, anti-oxidant, and anti-bacterial	*A*. *citrodora* *(L. citriodora)*	Geranial, neral, flavonoid, phenol, 1,8-cineole, limonene, and citral	[124,125,126,127,128,129,130,131,132,133]
*Origanum majorana* EO	Anti-melanoma, anti-bacterial, anti-oxidant, and anti-fungal	*O*. *majorana*	Terpinen-4-o1, L-α-Terpineol	[149,150]
*Artemisia capillaris* EO	Anti-melanogenesis, anti-cancer, and anti-oxidant	*A*. *capillaris*	Grass clumps	[118,156,157]
*Camellia japonica* seed EO	Anti-melanogenesis, anti-oxidant, anti-bacterial, anti-inflammatory, and skin barrier function	*C*. *japonica*	Seed (Hexamethylcyclotrisiloxane and octamethylcyclotetrasiloxane)	[160,161,162,163,164,165,168]
*Origanum syriacum* EO and *Origanum ehrenbergii* EO	Anti-melanogenesis, rheumatism maceration, and neuralgic treatment	*O*. *syriacum* and *O*. *ehrenbergii*	Terpenoids, quinones, and CRV	[57,169,170,171]
*Calocedrus formosana* EO	Anti-melanogenesis, anti-oxidant, anti-fungal, anti-inflammatory, and anti-cancer	*C* *. formosana*	α-Terpineol, Terpinen-4-o1, and thymol	[172,173,174,175,176,177]
*Melaleuca quinquenervia* EO	Anti-melanogenesis, anti-bacterial	*M* *. quinquenervia*	1,8-Cineole, α-Pinene, and α-Terpineol	[178,179,180]
*Cinnamomum cassia* EO	Anti-melanogenesis, anti-bacterial, anti-inflammatory, anti-diabetic, hypouricemic, and anti-fungal	*C. cassia*	Stem bark and twig (cis-2-methoxycinnamic acid and cinnamaldehyde)	[167,183,184,185,187,188,189]
Leaf of *Alpinia nantoensis* EO and rhizome of *Alpinia nantoensis* EO	Anti-melanogenesis, anti-cancer,	*A* *. nantoensis*	Leaf, rhizome, and stem (camphor, camphene, β-pinene, p-cymene, α-pinene, and D-limonene)	[191,192,193]
*Chrysanthemum boreale* MAKINO EO	Anti-melanogenesis, anti-inflammatory, anti-bacterial, skin regeneration, and wound healing	*C* *. boreale*	Flower	[194,196,197]
*Vitex negundo* EO	Anti-melanogenesis, anti-nociceptive, anti-convulsant, anti-inflammatory, and analgesic	*V* *. negundo*	leaves and roots (sesquiterpene and monoterpene)	[199,200,201,202,203,204]
*Achillea millefolium* EO	Anti-melanogenesis, alleviation of inflammatory, gastrointestinal disorders, hepatobiliary conditions, overactive cardiovascular, and respiratory infection	*A* *. millefolium*	Artemisia ketone, camphor, linalyl acetate, and 1,8-cineole	[206,207,208,209,210,211]
*Vetiveria zizanioides* EO	Anti-melanogenesis, anti-oxidants, antimicrobial, and anti-inflammatory	*V* *. zizanioides*	Roots (Cedr-8-en-13-ol and α-Amorphene)	[212,213,214,215,216,217]
*Eucalyptus camaldulensis* EO	Anti-melanogenesis, anti-bacterial, anti-termitic, and anti-oxidant	*E* *. camaldulensis*	Leaf (Eucalyptol and γ-Terpinene)	[220,221,222,223]
*Acorus macrospadiceus* EO	Anti-melanogenesis	*A*. *macrospadiceus*	Chavicol methyl ether and nootkatone	[228]
*Artemisia montana* Pampan EO	Wound healing, anti-diabetic, anti-inflammatory, and anti-oxidant	*A* *. montana*	β-caryophyllene, germacrene D, 1,8-cineole, and camphor	[238,239,240,241]
*Pistacia lentiscus* PO	Wound healing, anti-bacterial, anti-oxidants, and proliferation	*P*. *lentiscus* L.	Oleic, palmitic, linoleic, tocopherols, carotenoids, and anthocyanins	[243,244,245,246,247,248,249,250,251]
*Calophyllum inophyllum* PO	Anti-inflammatory, anti-microbial, anti-fungal, and wound healing	*C*. *inophyllum*	Calophyllolide, calophyllic acid, inophyllum, and polyphenols	[261,262,263,264,265]
*Parrotiopsis jacquemontiana* PO	Wound healing, anti-microbial, and anti-cancer	*P. jacquemontiana*	2, 6-dimethyl-8-oxoocta-2, 6-dienoic acid, syringol, and catechol	[266,267,268]
*Salvia aurea* L. EO	Wound healing	*S* *. aurea*	1,8-cineole, β-pinene, cis-thujone, and camphor	[276]
Polyphenol-enriched fraction of Rose plant oil distillation wastewater	Hyperproliferation inhibition, anti-HIV, anti-bacterial, anti-oxidant, anti-tussive, anti-diabetic, anti-inflammatory, and anti-plasmodial	*R*. *damascena* Mill. F. trigintipetala Dieck	Polyphenols, flavonoid	[232,277,278]
*Zanthoxylum bungeanum* Maxim EO	Hyperproliferation inhibition, anti-oxidants, anti-cancer, anti-inflammatory, anti-microbial, and anti-insecticidal	*Z*. *bungeanum*	D-Limonene, β-Myrcene	[286,287,288,289,290,291]
*Prunus armeniaca* EO	Hyperproliferation inhibition, anti-cancer, anti-oxidant, anti-microbial, anti-inflammatory, and anti-asthmatic,	*P* *. armeniaca*	Seeds (Benzaldehyde, benzoic acid, mandelonitrile)	[294,295,296,297,298,299,301]

Abbreviation: *C*. *limetta, Citrus limetta; B*. *dracunculifolia, Baccharis dracunculifolia; P. frutescens, Perilla frutescens; C*. *maxima, Citrus maxima; O. vulgare, Origanum vulgare; CRV, Carvacrol; S*. *sahendica, Satureja sahendica; M. chamomilla, Matricaria chamomilla; H. annuus, Helianthus annuus; M*. *arvensis, Mentha arvensis; R*. *officinalis, Rosmarinus officinalis; C. longa, Curcuma longa; A. argyi, Artemisia argyi; L*. *angustifolia, Lavandula angustifolia; Z*. *coreanum, Zanthoxylum coreanum; A. citrodora, Aloysia citrodora; L*. *citriodora, Lippia citriodora; O*. *majorana, Origanum majorana; A*. *capillaris, Artemisia capillaris; C*. *japonica, Camellia japonica; O*. *syriacum, Origanum syriacum; O*. *ehrenbergii, Origanum ehrenbergii; C*. *formosana, Calocedrus formosana; M*. *quinquenervia; Melaleuca quinquenervia; C. cassia; Cinnamomum cassia; A*. *nantoensis, Alpinia nantoensis; C*. *boreale, Chrysanthemum boreale; V*. *negundo, Vitex negundo; A*. *millefolium, Achillea millefolium; V*. *zizanioides, Vetiveria zizanioides; E*. *camaldulensis, Eucalyptus camaldulensis, A. macrospadiceus, Acorus macrospadiceus; A*. *montana, Artemisia montana; P*. *lentiscus, Pistacia lentiscus; C*. *inophyllum, Calophyllum inophyllum; P. jacquemontiana, Parrotiopsis jacquemontiana; S*. *aurea, Salvia aurea; R*. *damascena, Rosa damascene; Z*. *bungeanum, Zanthoxylum bungeanum; P*. *armeniaca, Prunus armeniaca*.
